# Determinants of metastatic competency in colorectal cancer

**DOI:** 10.1002/1878-0261.12018

**Published:** 2017-01-03

**Authors:** Daniele V. F. Tauriello, Alexandre Calon, Enza Lonardo, Eduard Batlle

**Affiliations:** ^1^ Institute for Research in Biomedicine (IRB Barcelona) The Barcelona Institute of Science and Technology Spain; ^2^ Hospital del Mar Medical Research Institute (IMIM) Barcelona Spain; ^3^ Institució Catalana de Recerca i Estudis Avançats (ICREA) Barcelona Spain

**Keywords:** cancer immunology, cancer stem cells, clonal diversity, combination therapy, dormancy, Heterogeneity, immunotherapy, stroma, TGF‐beta, tumour microenvironment

## Abstract

Colorectal cancer (CRC) is one of the most common cancer types and represents a major therapeutic challenge. Although initial events in colorectal carcinogenesis are relatively well characterized and treatment for early‐stage disease has significantly improved over the last decades, the mechanisms underlying metastasis – the main cause of death – remain poorly understood. Correspondingly, no effective therapy is currently available for advanced or metastatic disease. There is increasing evidence that colorectal cancer is hierarchically organized and sustained by cancer stem cells, in concert with various stromal cell types. Here, we review the interplay between cancer stem cells and their microenvironment in promoting metastasis and discuss recent insights relating to both patient prognosis and novel targeted treatment strategies. A better understanding of these topics may aid the prevention or reduction of metastatic burden.

**Abbreviations**
AJCC: American Joint Committee on Cancer
CAF: cancer‐associated fibroblast
CMS: consensus molecular subtype
CRC: colorectal cancer
CRC‐SC: colorectal cancer stem cell
ctDNA: circulating tumour DNA
CTL: cytotoxic T lymphocyte
EMT: epithelial–mesenchymal transition
EndMT: endothelial–mesenchymal transition
HSC: hepatic stellate cell
ISC: intestinal stem cell
ITH: intratumoral heterogeneity
MDSC: myeloid‐derived suppressor cell
Met‐SC: metastatic stem cell
MSC: mesenchymal stem cell
MSI: microsatellite‐instable
SC: stem cell
TAM: tumour‐associated macrophage
TAN: tumour‐associated neutrophil
TME: tumour microenvironment
TNM: tumour, lymph node, metastasis
UPR: unfolded protein response

## 1 Introduction

Colorectal cancer (CRC) is one of the most frequent types of cancer worldwide, accounting for approximately 10% of all new cancer cases and 8.5% of all cancer deaths (Torre *et al*., 2015). Whereas the vast majority of primary cancers can be extirpated through surgical resection, only a fraction of the patients diagnosed with overt metastatic disease can be cured by surgery. About 20% of the patients with CRC present with metastasis at the time of diagnosis (stage IV). In addition, 35–45% of the patients with localized disease (stages II and III) succumb to recurrence within 5 years after surgery. Most of these relapses occur as metastases and are caused by residual tumour cells that have spread to distant organs prior to surgery. Clearly, current systemic therapies fail to eliminate latent disseminated tumour cells and are similarly ineffective in treating growing metastases, offering survival benefits of only a few months.

Metastasis is the spread of cancer to a distant organ, which in the case of patients with CRC involves mainly the liver and lungs. As described elsewhere (Massague and Obenauf, 2016; Oskarsson *et al*., 2014; Valastyan and Weinberg, 2011), to gain metastatic competence, cancer cells require the capacity to invade the surrounding tissues, survive in the circulation, colonize the foreign organ and eventually resume growth. Metastasis is an inefficient process owing to the fact that most tumour cells fail to acquire the necessary abilities to regenerate a tumour at a distant site (Massague and Obenauf, 2016; Oskarsson *et al*., 2014). Over the past few years, the main determinants of metastatic competence in CRC have begun to be characterized. In the absence of mutations that associate with the process of metastasis in CRC, it has become increasingly clear that the regeneration of the tumour in a foreign organ is tightly bound to the acquisition of a stemlike phenotype by cancer cells. These metastatic stem cells adopt multiple phenotypes and behaviours and critically depend on their interaction with the microenvironment to migrate, survive in the circulation and thrive in a foreign organ.

In Section 2, we will review the genetics of CRC development, to then discuss both the evidence that supports the notion of hierarchical organization throughout CRC progression and the ensuing implications in Section 5. We will also focus on the mechanisms involved in the multiple phenotypes and adaptations that tumour stem cells go through in the metastatic process. In Section 2.2, we will explore the current knowledge about the role of the tumour microenvironment in promoting and sustaining metastasis. This includes a dissection of the cell types and niches that support the survival and maintenance of metastatic stem cells, and an analysis of the ways that stromal features can improve disease prognosis. In Section 3, we will discuss the complexities and limitations imposed on clinical practice by the heterogeneous nature of both epithelial and stromal compartments. Finally, we will indicate how these emerging concepts are informing, and slowly transforming, therapeutic strategies to treat patients with metastatic disease.

## 2 Colon carcinogenesis

### 2.1 From normal mucosa to colorectal cancer

The epithelium of the normal colon undergoes continuous renewal. At the base of glandular invaginations of the colonic mucosa, called crypts, a pool of rapidly diving intestinal stem cells (ISCs) sustains the homoeostatic regeneration of the epithelium throughout a lifetime (Clevers, 2013). Over the past decade, signals that regulate ISC renewal and proliferation have been extensively characterized: WNT, EGFR/MAPK and NOTCH signalling promote the undifferentiated proliferative state of ISCs in the niche, whereas BMP and TGF‐beta signalling induce cytostasis and differentiation (Clevers, 2013).

The elevated division rate of ISCs increases their probability to acquire mutations during DNA replication (Vogelstein *et al*., 2013). Additional environmental factors such as lifestyle, diet and microbiota can also greatly influence the transformation of the epithelium (Bishehsari *et al*., 2014). The most common genetic events in CRCs are alterations that inactivate the tumour suppressor gene *APC*. This triggers the constitutive activation of WNT signalling and imposes a continuous stemlike self‐renewing state at the onset of tumorigenesis, giving rise to benign outgrowths of the epithelium known as adenomas. Genetic experiments performed in mouse models support the hypothesis that *Apc* mutation in ISCs represents the origin of intestinal polyps (Barker *et al*., 2008; Tetteh *et al*., 2016), although chronic inflammation or dysregulation of BMP signalling has been shown to help convert non‐stem cells into CRC‐initiating cells (Davis *et al*., 2014; Schwitalla *et al*., 2013).

A small fraction of adenomas become progressively aggressive through acquisition of additional driver mutations, which mainly affect three additional signalling pathways (Cancer Genome Atlas Network, 2012; Seshagiri *et al*., 2012): (a) the MAPK pathway is often hit by activating mutations in *KRAS*,* BRAF* or *PIK3CA* and provides cell autonomous mitogenic and pro‐survival stimuli to cancer cells; (b) the p53 pathway is inactivated by mutations in the eponymous protein, or less commonly in ATM, facilitating acquisition of genomic instability; and (c) the TGF‐beta pathway is frequently silenced by loss‐of‐function mutations in *TGFBR2*,* SMAD4*,* SMAD2* or *SMAD3,* which bypasses the suppressive effects of high TGF‐beta levels present in the tumour microenvironment (Fearon, 2011). Pioneer studies by Eric Fearon and Bert Vogelstein correlated these mutations with pathologically classifiable stages of adenoma malignancy and suggested a linear progression model, in which the compounding of the four mentioned pathway mutations associated with development of aggressive adenocarcinomas (Fearon and Vogelstein, 1990).

Acquisition of these mutations is a slow process, and consequently, the development of invasive CRC often takes decades (Jones *et al*., 2008; Vogelstein *et al*., 2013). Of note, the linear progression model based on four stepwise genetic alterations represents a simplification, as not every tumour carries genetic alterations in these four pathways or develops through the equivalent sequence of events. Moreover, full‐blown CRCs have a riche and complex mutational landscape that expands well beyond mutations in the four driver pathways (Cancer Genome Atlas Network, 2012; Seshagiri *et al*., 2012). Due to the acquisition of chromosomal instability or defects in the DNA mismatch repair system, tumours accumulate hundreds or even thousands of genetic alterations. Some of these are passenger mutations, as they do not confer advantages to tumour cells, but others drive the biology of the cancer and therefore give selective advantage. Beyond the context of the linear progression model, the role of many of these mutations remains poorly understood. Together, these issues of complexity and heterogeneity impinge upon the functional analysis of CRC and complicate the development and application of therapeutic approaches.

### 2.2 Progression to metastasis

As described above, the mutations that drive CRC progression affect the signalling pathways that regulate ISC behaviour, endowing cancer cells with self‐renewal and growth capacity, independently of crypt niche signals. Evidence obtained from the analysis of patient‐derived and CRISPR‐engineered CRC organoids has led to the hypothesis that acquisition of mutations in the four linear progression model pathways may be sufficient to facilitate the growth of tumour cells in unfavourable environments such as those encountered in foreign tissues (Drost *et al*., 2015; Fujii *et al*., 2016; Matano *et al*., 2015). Yet, while most CRCs carry genetic alterations in several of these four driver pathways, metastasis is a relatively inefficient process. This suggests that additional bottlenecks and dependencies limit the extent of tumour spread.

It is worth considering that most CRCs are invasive at the time of diagnosis and, therefore, have had the opportunity to shed cells into circulation for months or longer. When disseminating CRC cells enter the portal circulation, they are transported to the liver sinusoids within minutes and can home into the liver parenchyma because of vessel fenestration. In the case of pulmonary metastases, CRC cells must first reach the general circulation and then infiltrate the lung parenchyma. It has been reported that this process requires active killing of lung capillary cells in a mechanism that involves the hormone PTHLH (Urosevic *et al*., 2014) and possibly necroptosis (Strilic *et al*., 2016). The capacity of disseminated CRC cells to infiltrate other organs such as the brain is less well characterized and may involve co‐option of invasion mechanisms described for other tumour types such as breast cancer (Valiente *et al*., 2014).

**Figure 1:**
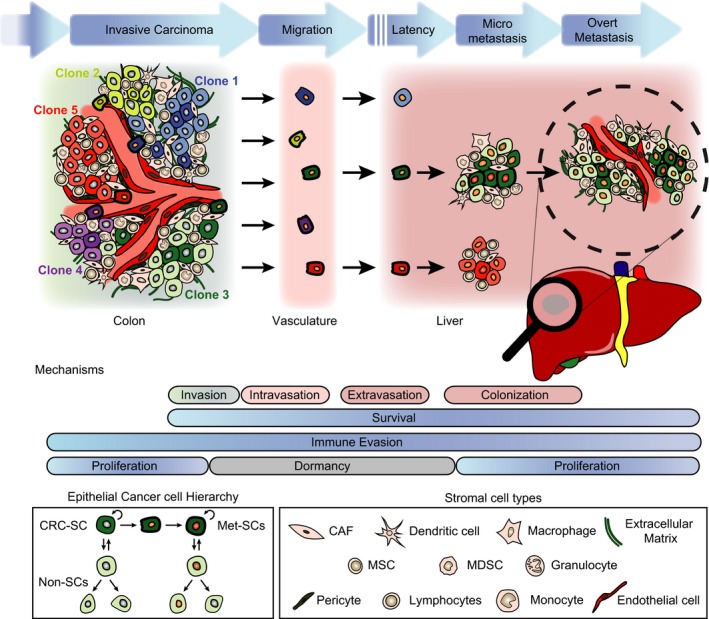
Types of heterogeneity underlying the process of CRC metastasis. Schematic representation of a primary tumour with clonal diversity (represented by different colours), tumour microenvironment (different cell shapes) and cellular hierarchy: colorectal cancer stem cells (CRC‐SCs) are drawn with darker colours than non‐stem cells (non‐SCs). In addition, metastatic stem cells (Met‐SCs) are represented by thicker outlines. There are distinct steps (blue arrows) along the metastatic process, each with attrition rates: survival in the vasculature during migration, overcoming mechanisms of latency and managing to establish an overt metastatic colony. During these events, interactions with the microenvironment that promote survival, immune evasion, dormancy/proliferation and stemness are thought to determine outcome. Below: legends depict a basic scheme of cellular hierarchy and the wealth of stromal cell types.

Work in experimental models has shown that the rate‐limiting step in the metastasis process is the capacity of circulating tumour cells to colonize the foreign organ (Massague and Obenauf, 2016; Obenauf and Massagué, 2015). Most tumour cells that survive in circulation and manage to infiltrate a distant organ will die for reasons that remain incompletely understood but that may include recognition and killing of tumour cells by the innate and adaptive immune system (Collignon *et al*., 2004; Strauss and Thomas, 2010). Those tumour cells that survive and adapt to the new environment can generate an overt metastasis (Fig. 1). However, not all venturing cancer cells that have successfully pervaded a distant site necessarily have the competency to establish a thriving colony in these hostile environments. Frequently, they remain latent for months to years before resuming growth (Sosa *et al*., 2014). Below we discuss tumour cell‐intrinsic and cell‐extrinsic aspects that facilitate metastatic colonization in CRC.

## 3 Colorectal cancer stem cells and their role in metastasis

Besides (genetic) diversity between patients, no individual CRC is a uniform, clonal mass of cancer cells: any given tumour consists of multiple cell populations with varying levels of phenotypic and genetic heterogeneity. Intratumoral heterogeneity (ITH) is a central concept to understand the phenomena of metastatic progression and therapeutic resistance. Three factors contribute to ITH: the hierarchical organization of cell lineages (Sections 3.1 to 3.3), their clonal diversification (Section 3.4) and the microenvironment (Section 4).

### 3.1 Hierarchical organization of cancer cells

More than 30 years ago, it was already proposed that the phenotypic diversity in cancers could arise from spontaneous differentiation of tumour cells (Pierce and Speers, 1988). The concept, originally developed by G. Barry Pierce in the 1970s, states: ‘[carcinomas] are composed of a mixture of malignant stem cells, which have a marked capacity for proliferation and a limited capacity for differentiation under normal homeostatic conditions, and of the differentiated, possibly benign, progeny of these malignant cells’ (Pierce and Speers, 1988). This hypothesis was long ignored, yet several laboratories have recently put forward evidence to support that CRC complies with this concept. Of note are studies by the groups of Ruggero de Maria (Ricci‐Vitiani *et al*., 2007), John Dick (O'Brien *et al*., 2007) and Michael Clarke (Dalerba *et al*., 2007), wherein each identified a population of tumour cells within human CRCs that has the rare capacity to propagate the disease upon inoculation into immunodeficient mice. Relaunching the idea that CRCs are organized through a hierarchy of cells with distinct tumorigenic potential, they named this population ‘tumour‐initiating cells’.

Subsequent investigation showed that tumour‐initiating cells (also referred to as CRC stem cells or CRC‐SCs) reside at the apex of a hierarchy of tumour cells. They self‐renew, display long‐term proliferation potential and are capable of initiating tumours when inoculated into mice. CRC‐SCs cells express a gene programme that to some extent overlaps with that of normal ISCs, and their progeny can undergo differentiation towards a phenotype similar to that of the normal mucosa. Differentiation coincides with loss of tumorigenic potential (Dalerba *et al*., 2011; Kreso *et al*., 2014; Merlos‐Suarez *et al*., 2011; Vermeulen *et al*., 2008, 2010). Furthermore, the existence of stem cell hierarchy in CRC has been backed by lineage tracing studies in adenomas (Kozar *et al*., 2013; Schepers *et al*., 2012) and by studies of fate‐mapping analysis using lentiviral marking of individual tumour cells (Dieter *et al*., 2011; Kreso *et al*., 2012).

The balance between stemness and differentiation in CRC depends upon the same pathways that regulate normal ISCs, including WNT (Vermeulen *et al*., 2010), BMP (Lombardo *et al*., 2011) and NOTCH (Lu *et al*., 2013) signalling. Many of these signals are provided by cells of the tumour stroma, a finding that somewhat contradicts the hypothesis that CRC progresses through gain of ISC niche independency by acquired mutations. Yet, it is worth considering that not every CRC carries alterations in all four driver pathways (WNT, EGFR, TGF‐beta/BMP and p53) and thus may still depend on stromal factors for further progression. As is discussed below, additional stromal‐derived cytokines and growth factors present at the sites of invasion can further promote self‐renewal of CRC‐SCs.

### 3.2 Tumour cell phenotypes at the invasion front

Because CRC‐SCs have both self‐renewal and tumour‐initiating capacity, they likely represent (or give rise to) the so‐called metastatic stem cells (Met‐SCs; Fig. 1), that is the cell of origin of metastasis (Oskarsson *et al*., 2014). Experimental evidence supports this hypothesis: the above‐mentioned study by the group of Hanno Glimm (Dieter *et al*., 2011) demonstrates that only cells that hold long‐term self‐renewing ability are capable of generating metastasis.

**Figure 2:**
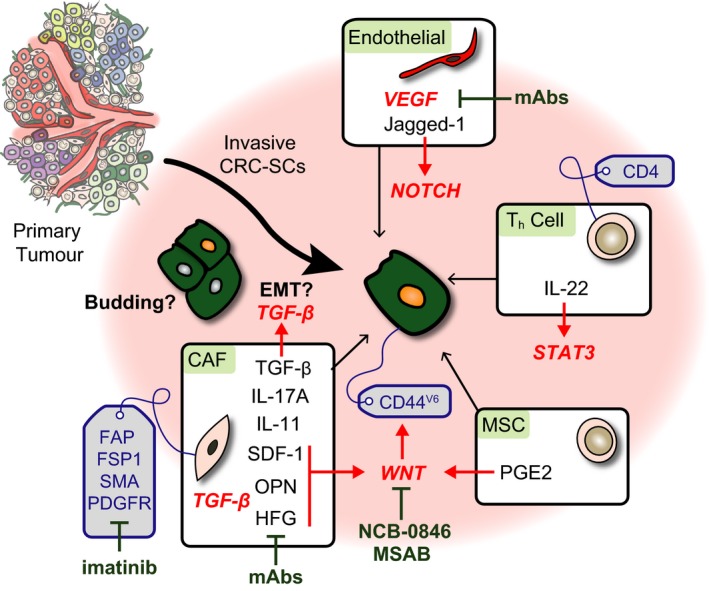
Mechanisms and therapeutic opportunities in early dissemination. In the centre is a cancer stem cell (CRC‐SC) that is in the process of invasion (which might involve EMT or tumour budding) or vascular migration. Depicted are various recently described interactions with stromal cells: proteins expressed by stromal cells are depicted in black, processes in bold, activated pathways in red and relevant cellular markers are written in blue (drawn inside grey labels). Agents or inhibitors that target pathways or proteins are drawn in green. See the main text for more details.

Most CRCs are a relatively disorganized mixture of stem‐ and differentiated‐like cells that reside into glandular structures reminiscent of the normal crypts (Merlos‐Suarez *et al*., 2011). Metastases often have an equivalent appearance (Merlos‐Suarez *et al*., 2011). However, whereas CRC‐SCs present in tumour glands are tumorigenic if isolated and xenografted into mice, naturally they will only be competent to generate metastasis if they first acquire phenotypic changes that enable their migration and extravasation. This process is thought to occur when cancer cells engage in communication with the adjacent tissues and the tumour stroma. A key example is the interaction with endothelial cells, which induce NOTCH signalling on cancer cells to facilitate transendothelial migration via the kinase ABL and RHO activity at invasion fronts (Sonoshita *et al*., 2011, 2015) (Fig. 2).

In the interest of defining Met‐SCs, there is a long‐standing focus on cancer cells at invasion fronts. A frequent finding in CRCs is the presence of tumour buds – small clusters of detached cancer cells at the invasive border. These budding cells typically possess attributes that help explain their invasive and migratory phenotype, including increased expression of genes involved in extracellular matrix degradation and in epithelial–mesenchymal transition (EMT) (Zlobec *et al*., 2010). Moreover, cancer cells present at invasion fronts and in tumour buds display increased accumulation of nuclear beta‐catenin (Brabletz *et al*., 1998), possibly marking stemness. Although most CRCs carry mutations that activate the WNT pathway constitutively, a number of signalling molecules emanating from stromal cells such as HGF (Vermeulen *et al*., 2010) and PGE2 (Li *et al*., 2012) have been shown to further elevate WNT signalling in adjacent areas (Fig. 2). In contrast, high levels of nuclear beta‐catenin at invasion fronts do not always correlate with tumour budding and various stem cell markers have been found in only a small fraction of budding cells, casting some doubt on their general representation of Met‐SCs (reviewed in Dawson and Lugli, 2015). Notwithstanding these caveats, tumour budding has consistently been linked to poor survival and is a prognostic factor poised to refine standard clinical risk assessment of early‐stage CRC (van Wyk *et al*., 2015).

Additional factors derived from the tumour microenvironment that help sustain invasiveness and self‐renewal of CRC‐SCs include IL‐22, expressed by a subset of T cells (Kryczek *et al*., 2014), and fibroblast‐derived IL‐17A (Lotti *et al*., 2013). Perhaps the best example connecting invasion and stemness in CRC is the work by Giorgio Stassi, de Maria and colleagues, who found that a subpopulation of CRC‐SCs expressing the surface marker CD44‐v6 is present at invasion fronts and gives rise to metastatic lesions in experimental models (Todaro *et al*., 2014). CD44‐v6 is required for cell migration, and its expression is increased by factors secreted by stromal cells such as HGF, OPN and SDF‐1 (Todaro *et al*., 2014). Together with the above‐mentioned WNT‐stimulating mechanisms, these data imply that the microenvironment instructs the formation of migratory CRC‐SCs, or putative Met‐SCs (Fig. 2).

Following local invasion, CRC cells that enter the vasculature are termed circulating tumour cells (CTCs). From a theoretical perspective, both CRC‐SCs and non‐SCs may be able to enter the circulation. Yet, few studies have assessed the heterogeneity of CTCs in regard to their stem cell properties, such as CD133 expression (Iinuma *et al*., 2011). Still, CTCs have attracted considerable attention for their diagnostic potential, although many challenges – including heterogeneity – impede their robust detection and thus exploitation (reviewed in Hardingham *et al*., 2015). In contrast, analysing mutations in circulating tumour DNA (ctDNA) appears to be a more straightforward and robust method to detect residual disease and thus to assess risk of relapse after therapy in patients with localized disease (Tie *et al*., 2016).

### 3.3 Slow‐proliferating and dormant tumour cells

As described in the introductory section, metastasis often develops after a period of latency, which in CRC typically ranges up to 5 years. Latency is the consequence of disseminated cells remaining in a dormant state at distant sites. The current therapeutic strategy to eliminate these residual cells is treatment with standard chemotherapy, which has a limited benefit for the patients. Indeed, dormant and slow‐proliferating cells are largely resistant to chemotherapy, as this typically targets rapidly proliferating cells. In addition, dormant cells remain in a particular state of resilience against cell death‐inducing signals and may even be protected against attacks of the immune system (Ghajar, 2015; Malladi *et al*., 2016; Massague and Obenauf, 2016). Metastatic latency likely includes two distinct mechanisms: population dormancy, a condition in which tumour cell proliferation and death are balanced, thus leading to micrometastatic lesions that do not expand; or a state of cellular quiescence or temporary mitotic arrest (Ghajar, 2015; Massague and Obenauf, 2016).

The characterization of these mechanisms is of key importance, as a better understanding offers opportunities to cure patients by eliminating the residual disease (or preventing its outgrowth) before appearance of overt metastasis. Although progress in this area has been made for other cancer types (for excellent reviews on this topic, see Ghajar, 2015; Massague and Obenauf, 2016), these processes remain poorly elucidated in CRC. Concerning the latter mechanism, fate‐mapping experiments with xenografts support the existence of slow‐proliferating cells in the tumour bulk. Specifically, not all tumour‐initiating cells responsible for secondary and tertiary transplants had detectably contributed to primary xenografts, implying the existence of dormant cells that can reactivate in tumour re‐initiation (Dieter *et al*., 2011). Through a similar strategy, such dormant or minor clones were found to gain predominance upon chemotherapy treatment (Kreso *et al*., 2012).

Whereas the origin, identity and regulation of dormant cell populations in CRCs remain unclear, advances in the understanding of cellular diversity of the normal intestinal epithelium may provide some clues. In the crypts, most cells positive for ISC marker Lgr5 proliferate at high rates and are therefore sensitive to treatment with radio‐ and chemotherapy (Metcalfe *et al*., 2014; Yan *et al*., 2012). Douglas Winton and colleagues showed that a subset of Lgr5^+^ cells that are differentiating towards secretory lineages have slow proliferation kinetics and are relatively chemoresistant (Buczacki *et al*., 2013). Although these cells are not clonogenic in homoeostasis, they regain stemness and contribute to regenerating the epithelium after ISC pool depletion by treatment with cytostatic drugs. Likewise, lineage tracing experiments in similar settings have shown that differentiated cells of the absorptive and secretory lineage can undergo a process of dedifferentiation and repopulate the ISC niche, gaining self‐renewal capacity (van Es *et al*., 2012; Tetteh *et al*., 2015, 2016). Thus, differentiated cells constitute a reservoir of facultative stem cells in normal mucosa. Given the other parallels with CRC, such as cellular hierarchy, it would be clinically relevant to test whether these concepts of (population) dormancy and plasticity also apply to cancer and metastasis. For example, do latent metastatic cells share characteristics – other than chemoresistance – with quiescent crypt cells? And does dedifferentiation facilitate CRC recurrence after treatment?

### 3.4 Linking clonal diversity to cancer stem cell architecture

As a result of genomic instability, cancers acquire hundreds of genetic and epigenetic alterations that impose distinct phenotypes and fates on tumour cells, potentially leading to clonal expansion. This evolutionary phenomenon is the basis for the striking capacity of cancer to adapt to different environments, colonize foreign organs and resist therapy (Boland and Goel, 2005; Nowell, 1976; Swanton, 2012). In CRC, a Big Bang‐like model has been proposed in which tumours go through extensive clonal diversification at early stages, with little indication for stringent selection or clonal expansion. Additionally, malignant behaviour appears to be determined early, and can be inferred from analysing subclone intermixing events (Sottoriva *et al*., 2015). From this initial genetic diversity, artificial selection pressure in the form of chemotherapy might select for the rise of pre‐existing resistant clones (reviewed in Greaves and Maley, 2012). For instance, minor populations of KRAS mutant cells already present in the primary CRC will expand upon anti‐EGFR therapy, leading to resistant relapses (Diaz *et al*., 2012).

At the conceptual level, the phenotypic heterogeneity resulting from tumour cell hierarchy and clonal diversity is not necessarily independent. CRC‐SCs likely represent the unit of clonal selection, as mutations occurring in more differentiated cells may have a lower chance of being selected given the relatively short life span of this population (reviewed in Greaves and Maley, 2012; Kreso and Dick, 2014; Valent *et al*., 2012). A particularly interesting aspect that has been seldom explored is how the genotype affects cellular hierarchy. The WNT (Vermeulen *et al*., 2010), PI3K (Tenbaum *et al*., 2012), BMP/TGF‐beta (Lombardo *et al*., 2011) signalling pathways all affect self‐renewal and differentiation capacity of CRC stem cells, and thus, mutations in these pathways likely regulate the frequency of CRC‐SCs. It is thus possible that successive accumulation of genetic alterations in these pathways increases frequencies of CRC‐SCs, progressively increasing the probability of mutation being selected and thus accelerating evolution, up to a point where full compound mutant tumours contain abundant, variegated populations of CRC‐SCs with limited capacity to produce differentiated progeny. Testing this hypothesis will require analysing how the tumour hierarchy changes in distinct mutational backgrounds.

Although clonal diversity has been linked to therapeutic resistance, somatic evolution and increased metastatic competency, sequencing of primary CRC and metastases has revealed no specific genetic alterations associated with tumour dissemination *per se* (Jones *et al*., 2008; Mlecnik *et al*., 2016b). While equivalent studies analysing epigenetic marks have not been performed, the similarities between primary tumour and metastasis suggest that nongenetic factors might be of particular relevance in this process.

## 4 The microenvironment during progression to metastasis

**Figure 3:**
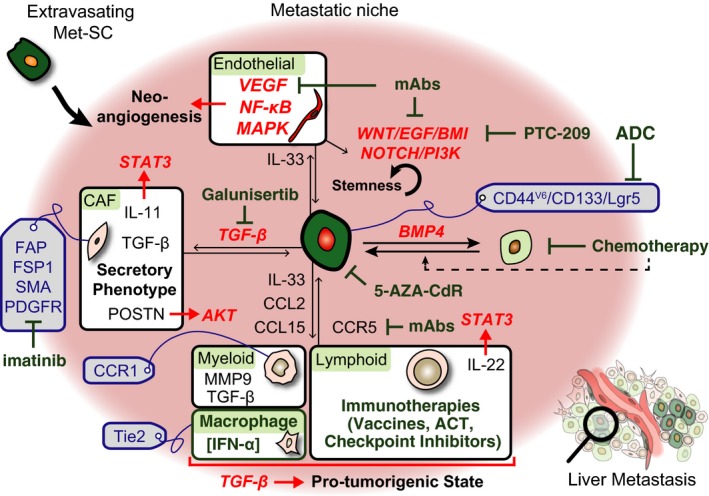
Mechanisms and therapeutic opportunities in the metastatic setting. In the centre is a metastatic stem cell (Met‐SC) that is in the process of colonizing the liver. Depicted are various recently described interactions with stromal cells: proteins expressed by stromal cells are depicted in black, processes in bold, activated pathways in red and relevant cellular markers are written in blue (drawn inside grey labels). Agents, inhibitors or therapeutic strategies that target pathways or proteins are drawn in green. See the main text for more details.

Heterogeneity between patients and within the CRC epithelial compartment already raises significant challenges and opportunities for patient diagnosis and treatment. Yet, further augmenting the complexity, ITH also includes the numerous additional cell types that permeate the tumour and are collectively referred to as stroma or tumour microenvironment (TME). Stromal‐associated functions are linked to all steps of cancer progression and metastasis, and a picture has emerged in which cancer cells and the stroma cross‐communicate and co‐evolve during cancer progression (Hanahan and Coussens, 2012; Quail and Joyce, 2013). Conceptually, transformed cancer cells strongly change the nature and composition of the stroma, to the point that this altered microenvironment forms an adapted niche, providing protection and stimulation, essentially fostering cancer stem cells (Fig. 3).

With the emergence of the TME as a critical player in cancer progression, an increasing effort goes into the characterization of specific stromal cell populations that are held responsible for tumour malignization and metastatic colonization. Several mechanisms have issued from this endeavour, providing potential targets for the design of new anticancer therapies.

### 4.1 Cancer‐associated fibroblasts

Cancer‐associated fibroblasts (CAFs) are a heterogeneous group of fibroblasts that are redirected towards tumour promotion. As in other cancer types, CRC‐associated fibroblasts differ from neighbouring normal fibroblasts and express specific markers including alpha‐smooth muscle actin (alpha‐SMA), fibroblast surface protein (FSP‐1) and fibroblast‐activated protein (FAP) (reviewed in Calon *et al*., 2014). CAFs provide CRC cells with an array of cytokines that promote cancer cell survival and tumour initiation (Kalluri, 2016). Mechanistically, co‐inoculated CAFs enhance *in vivo* tumour growth of cancer cells more than normal colon fibroblasts, and soluble factors secreted by the former increase self‐renewal and migration of epithelial cancer cells to a greater extent than those secreted by the latter (Berdiel‐Acer *et al*., 2014). In addition, gene expression signatures derived from CAFs correlate with poor outcome in colorectal cancer (Becht *et al*., 2016; Calon *et al*., 2015; Herrera *et al*., 2013; Isella *et al*., 2015). Thus, CAFs constitute an important cell population in the TME and provide a permissive niche for cancer progression (Figs 2 and 3).

TGF‐beta has been linked to poor prognosis in CRC as an independent biomarker across AJCC (American Joint Committee on Cancer) stages, and ligand expression levels correlate with TGF‐beta‐activated stromal cells that secrete a cocktail of additional pro‐metastatic factors (Calon *et al*., 2012) (Fig. 3). Although TGF‐beta activates a wide range of tumour stroma cell types (Pickup *et al*., 2013), CAFs are the main contributors to the association of stromal TGF‐beta‐driven programmes with poor clinical outcome in CRC, suggesting a predominant role of TGF‐beta‐activated CAFs during progression to metastasis (Calon *et al*., 2015). These activated CAFs express several known pro‐metastatic secreted factors including angiopoietin‐like‐4 (ANGPTL‐4) (Padua *et al*., 2008), connective tissue growth factor (CTGF) (Kang *et al*., 2003), tenascin C (TNC) (Oskarsson *et al*., 2011) – functionally described in breast cancer – and periostin (POSTN), a promoter of metastatic growth in colon cancer that increases cell survival via the AKT pathway (Bao *et al*., 2004). In addition to these factors, we found that TGF‐beta‐activated CAFs secrete interleukin‐11 (IL‐11), which leads to enhanced STAT‐3‐dependent survival and initiation of metastatic cancer cells (Calon *et al*., 2012). To support the role of this cytokine in CRC progression, an IL‐11 antagonist was shown to reduce both proliferation and invasive capacity of CRC cells (Putoczki *et al*., 2013). A recent study described an additional function for TGF‐beta in promoting liver metastasis by the adhesion of cancer cells to CAFs, followed by their subsequent codissemination to the metastatic site (Gonzalez‐Zubeldia *et al*., 2015).

Besides the above‐mentioned TGF‐beta targets, also modifiers of TGF‐beta signalling may be therapeutically relevant in the context of CAFs and liver metastasis. Under physiological condition, hepatic stellate cells (HSCs) are maintained unresponsive to TGF‐beta through degradation of TGF‐beta receptor type‐2, by a process involving IQGAP1 (IQ motif‐containing GTPase‐activating protein). During cancer progression, paracrine signalling emanating from cancer cells decreases IQGAP1 expression in HSCs, which promotes HSC activation into myofibroblasts as well as metastatic outgrowth (Liu *et al*., 2013). These data indicate that CRC‐SCs are capable of initiating the formation of a metastatic niche by reprogramming resident mesenchymal cells into CAFs. Furthermore, TGF‐beta signalling in HSCs was shown to be modulated by platelet‐derived growth factor receptor (PDGFR)‐alpha, leading to paracrine effects on CRC cell proliferation and migration (Liu *et al*., 2014). This adds to the rationale for the inhibition of the PDGFR signalling pathway with imatinib, a compound targeting tyrosine kinases including PDGFRs. This drug was previously proposed as a therapeutic agent in CRC for its effects on impairing migration of bone marrow‐derived mesenchymal stem cells (MSCs) to the tumour site, resulting in decreased metastatic intake (Shinagawa *et al*., 2013). Incidentally, MSCs have also been reported to be differentiated into CAF‐like cells in the TME (Shinagawa *et al*., 2010).

### 4.2 Endothelial cells

The stromal niche surrounding cancer cells comprises a constantly developing network of blood vessels, supplying the growing tumour with oxygen and nutrients. Secreted factors produced in the tumour stimulate proliferation and survival of endothelial cells, thus enhancing angiogenesis. Tumour‐associated angiogenesis gives rise to abnormal blood vessels characterized by a chaotic network, excessive branching, decreased pericyte coverage and leakiness (Dudley, 2012). The latter has been associated with metastasis and poor prognosis in patients with CRC (Yonenaga *et al*., 2005).

VEGFA is a key regulator of endothelial cell proliferation in most human tumours, inducing the MAPK/ERK signalling pathway. Tumoral VEGFA expression correlates with invasiveness, increased vascular density and progression to metastasis. Accordingly, bevacizumab, a monoclonal antibody against VEGFA and an inhibitor of angiogenesis, has been shown to increase survival of patients with stage IV CRC when combined with chemotherapy (Ferrara and Adamis, 2016; Hurwitz *et al*., 2004; Mathonnet, 2014) (Figs 2 and 3). Besides blocking neo‐angiogenesis, the clinical benefit of a dual‐targeting approach may involve the reprogramming of tumour‐associated blood vessels into normalized blood vessels by bevacizumab, decreasing vessel permeability and improving anticancer drug bioavailability into the tumour mass (Jain, 2005).

Interleukin‐33 (IL‐33), a cytokine secreted by endothelial and epithelial cells to activate NF‐κB and MAPK signalling, may also hold therapeutic interest for the reprogramming of endothelial cells and normalization of tumour vasculature in CRC. Tumour‐derived IL‐33 dramatically enhances neo‐angiogenesis by increasing endothelial cell proliferation, migration and differentiation into blood vessels to robustly increase metastatic spreading of CRC cells to the liver (Zhang *et al*., 2016). Conversely, blockade of IL‐33 signalling suppresses angiogenesis and reduces tumorigenesis (Maywald *et al*., 2015).

Endothelial cells are a part of perivascular niches that have been reported to foster cancer stem cells (Butler *et al*., 2010). A mechanism in which endothelial cells directly promote the formation of CRC‐SCs involves a soluble form of Jagged‐1 to activate NOTCH signalling in cancer cells (Lu *et al*., 2013) (Fig. 2). Another way in which endothelial cells might participate more directly in niche formation is their conversion into CAF‐like cells, through a TGF‐beta‐driven mechanism called endothelial–mesenchymal transition (EndMT). This process is associated with the upregulation of fibroblast‐specific protein‐1 (FSP‐1) and downregulation of the endothelial marker CD31 (Zeisberg *et al*., 2007). Although the existence of EndMT in CRC remains elusive, a similar mechanism was reported in response to inflammation in the colon (Rieder *et al*., 2011).

### 4.3 Immune cells

As the immune system can be a powerful weapon against tumorigenesis through coordinated elimination of aberrant cells, successful cancers find a way to circumvent tumour immunity; this is recognized as a hallmark of cancer (Hanahan and Weinberg, 2011). In fact, it has become increasingly clear that the immune system has a dual role in cancer, able to both suppress and promote cancer progression (Schreiber *et al*., 2011). Even if a thriving cancer suggests inhibition of the former and exploitation of the latter, there is evidence for a continued battle between the immune system and cancer cells. First, there is a positive prognostic value of the presence of infiltrating immune cells such as T cells (Galon *et al*., 2006) and dendritic cells (Gulubova *et al*., 2012), as well as high levels of gene signatures of active immune responses (Galon *et al*., 2006). Conversely, the presence in blood and tumour of immunosuppressive myeloid‐derived suppressor cells (MDSCs) (Solito *et al*., 2011; Sun *et al*., 2012) correlates with a poor prognosis. Second, cancers show signs of immunoselection, where the number of neoantigens is lower than expected based on mutation rates. This can be caused by T cell‐mediated killing of cells or clones expressing immunogenic neoantigens (Matsushita *et al*., 2012) or by loss of antigen expression or presentation (DuPage *et al*., 2012; Rooney *et al*., 2015).

Together, this indicates that evasion from antitumour immunity is only partial, local or reversible and that immunity is likely a key hurdle to overcome in the metastatic process. Relatedly, metastatic latency and population dormancy have been linked to immunosurveillance in a melanoma model, where (micro)metastasis growth was equilibrated by immunologic cancer cell killing (Eyles *et al*., 2010) (reviewed in Giancotti, 2013; Sosa *et al*., 2014). Although many of these important concepts are emerging for cancer in general (Vinay *et al*., 2015), less is known about the specific situation in CRC. Nevertheless, below are some recently described mechanisms that together have an important impact on the prospects of immunotherapies.

#### 4.3.1 Innate immune cells

Besides their role in innate immunity and the coordination of adaptive immune responses, several bone marrow‐derived myeloid cells have been linked to tumour progression (Taketo, 2009). Chemokine signalling may play an important role in their recruitment and subsequent communication with CRC cells and other TME residents, often promoting both cancer progression and metastasis (Itatani *et al*., 2016) (Fig. 3). Immature myeloid cells positive for C‐C chemokine receptor type 1 (CCR1) have been linked to CRC progression and liver metastasis (Kitamura *et al*., 2007, 2010), in a mechanism that involves the loss of *SMAD4* in CRC cells, triggering CCL15‐mediated recruitment of CCR1^+^ myeloid cells (Hirai *et al*., 2014; Itatani *et al*., 2013). Similar mechanisms link MDSCs to CRC progression through the action of CCL15 (Inamoto *et al*., 2016) or CCL2 (Chun *et al*., 2015).

Macrophages, one of the most abundant tumour‐infiltrating cell types, have been ascribed many tumour suppressor roles in CRC, ranging from direct cytotoxicity to orchestrating and sustaining adaptive responses (Braster *et al*., 2015). Besides this classical functional state (often called M1), alternative activation states such as M2 (also referred to as tumour‐associated macrophages or TAMs) have been described; these are characterized by mostly pro‐tumorigenic potential (reviewed in Mantovani *et al*., 2009). Polarization of macrophages is incompletely understood but appears to be plastic and largely dependent on the TME (reviewed in Braster *et al*., 2015). Recently, much attention has gone towards the clinical implication of the presence, plasticity and dual functions of TAMs (Braster *et al*., 2015; Mantovani and Allavena, 2015). For example, their accumulation at the liver metastatic periphery was recently shown to lend itself for exploitation, when a gene transfer strategy delivered interferon‐alpha to the TME via Tie2^+^ monocytes/macrophages in a mouse model for CRC metastasis, resulting in reduced tumour growth and enhanced survival (Catarinella *et al*., 2016) (Fig. 3).

Like macrophages, neutrophils (a class of granulocytes) have been found to undergo functional repolarization in the TME, a process that involves TGF‐beta (Fridlender *et al*., 2009) and converts them into a pro‐tumorigenic state (N2 vs. N1). Tumour‐associated neutrophils (TANs), like MDSCs, can be recruited to SMAD4‐mutant metastatic cancer cells by CCL15 and help promote lung colonization (Yamamoto *et al*., 2016). In addition, a recent report links neutrophils to cancer recurrence after surgery through a defence mechanism involving the release of neutrophil chromatin strands: neutrophil extracellular traps (Tohme *et al*., 2016). These may be possible explanations for the emergence of a high neutrophil‐to‐lymphocyte ratio as a potential poor‐prognosis marker for patients with CRC (Li *et al*., 2014).

#### 4.3.2 Adaptive immune cells

Specialized, adaptive immune responses heavily rely on cells from the lymphoid lineage, including T cells and B cells. Central mediators of adaptive responses are CD8^+^ cytotoxic T lymphocytes (CTLs) that recognize specific antigens. For their function, they require assistance and reinforcement from CD4^+^ T‐helper cells as well as from myeloid cells. CTL infiltration in CRC is a factor for good prognosis, and lack thereof indicates poor disease outcome (Galon *et al*., 2006; Naito *et al*., 1998; Ropponen *et al*., 1997), indicating that T cell‐mediated immune surveillance plays an important role in CRC metastasis. Therefore, microsatellite‐instable (MSI) cancers, bearing multiple mutations that can translate into aberrant (peptide) antigens, may be linked to good prognosis – in large measure – because of effective adaptive immune responses (Kloor and von Knebel Doeberitz, 2016; Rooney *et al*., 2015).

A growing number of recent therapeutic strategies aim to enhance existing T‐cell responses, and early results point to successes mainly in MSI cancers (see Section 5.4). Besides reprogramming neutrophils, TGF‐beta has been shown to repolarize various other immune cell types, including natural killer cells, dendritic cells, macrophages and T cells (Flavell *et al*., 2010), and to directly inhibit T‐cell mechanisms (Chen *et al*., 2005; Thomas and Massague, 2005; Yang *et al*., 2010; Zhang *et al*., 2005). Moreover, dendritic cells, MDSCs and tumour‐associated macrophages are all, like CAFs, sources for stromal TGF‐beta (Gabrilovich and Nagaraj, 2009), indicating a mechanism in which TGF‐beta reprogrammes a variety of stromal cells, amplifying its own signal, and impeding anticancer immune responses in a concerted way.

### 4.4 Heterogeneity of the TME as a tool to stratify patients with CRC

As described above, an understanding of the extent of intra‐ and intertumour variegation helps frame the limited accuracy of the generic AJCC staging system in predicting disease outcome, and has provided a number of additional parameters for prognostic use. Numerous findings underscore the prognostic value of immune cells in the tumour stroma during cancer progression. As mentioned above, the work by Jérôme Galon and colleagues showed that an index evaluating the type and localization of immune cells (Immunoscore) can predict disease outcome. Indeed, they demonstrated a stronger prognostic power than the classical TNM (tumour, lymph node, metastasis) system (Galon *et al*., 2006, 2014; Mlecnik *et al*., 2016a,b).

Furthermore, since the development of DNA microarray technology, there has been a growing interest in refining CRC patient stratification with unbiased molecular classification based on gene expression profiles (Blanco‐Calvo *et al*., 2015; Golub *et al*., 1999). A series of recent studies have identified between three and six molecular subtypes of CRCs associated with distinct outcome and response to treatment (Budinska *et al*., 2013; De Sousa E Melo *et al*., 2013; Marisa *et al*., 2013; Roepman *et al*., 2014; Sadanandam *et al*., 2013; Schlicker *et al*., 2012). To consolidate the various classifiers, a consortium of experts in the field of molecular classification integrated these molecular stratifications of CRC into four consensus molecular subtypes (CMS) (Guinney *et al*., 2015). CMS1 includes most of the MSI and CpG island methylator phenotype (CIMP)‐high CRCs, whereas CMS2, CMS3 and CMS4 are generally chromosome‐instable tumours displaying roughly equivalent genotypes yet distinct expression profiles. CRCs belonging to CMS2 express a gene programme that suggests elevated WNT/MYC activation, and thus, they may represent the canonical class of CRC. CMS3 cancers express signatures that reflect particular metabolic reprogramming, and those of CMS4 display elevated expression of mesenchymal genes.

Importantly, CMS4 is associated with poor outcome. Whereas mesenchymal gene expression was initially attributed to cancer cells undergoing EMT, two studies revealed that the signatures that identified patients with the worst outcome were in fact of stromal origin (Calon *et al*., 2015; Isella *et al*., 2015). Recent analysis also suggests that the gene expression profile of the CMS4 subtype reflects immunosuppression (Becht *et al*., 2016). Importantly, the power of stromal gene signatures to predict disease relapse outperforms both the classical AJCC staging system and the consensus molecular stratification of patients (Calon *et al*., 2015; Isella *et al*., 2015). As mentioned in earlier sections, further investigation within these prognostic stromal gene signatures identified a prominence of TGF‐beta target genes expressed by CAFs in the most aggressive tumours, which are promising poor‐prognosis biomarkers that can be assessed using either transcriptomic or immunohistochemical techniques (Calon *et al*., 2012, 2015). Altogether, these data suggest that stromal evaluation will greatly benefit upcoming patient classification systems and may translate to better clinical assessment of the disease, while also providing new avenues for therapeutic intervention.

## 5 Specific targeting of cell types to treat metastasis

For many years, the standard of care for advanced disease had been 5‐fluorouracil – commonly supplemented with folinic acid – which only conferred a marginal survival advantage. Somewhat more encouraging results emerged from the addition of oxaliplatin or irinotecan to the regimen (FOLFOX or FOLFIRI, respectively) both in metastatic disease (Douillard *et al*., 2000; de Gramont *et al*., 2000) and, as adjuvant therapy, in some stage II and most stage III CRC patients (André *et al*., 2009; Van Cutsem *et al*., 2016). However, these systemic chemotherapies in effect indiscriminately kill proliferative cells and their therapeutic index is limited for many patients. Moreover, this approach neither targets dormant CRC‐SCs nor offers an answer to resistance or stromal mitigation. Understanding intratumoral heterogeneity and the biology of the different cell types that populate the tumour is guiding the development of new therapeutic strategies to treat advanced disease.

### 5.1 Targeted therapies

Standard systemic chemotherapy is increasingly combined with targeted treatments that eliminate specific dysregulated pathways crucial for cancer growth or survival. For example, inhibitors of EGFR signalling such as cetuximab and panitumumab improve survival in patients with CRC (Van Cutsem *et al*., 2016) (Fig. 3). Unfortunately, these therapies are met with both intrinsic and acquired resistance in the vast majority of cases, often involving mutations downstream to EGFR (including in *KRAS* and *BRAF* genes), but also in other mitogenic protein kinase receptor signalling pathways (Bertotti *et al*., 2015).

As additional targeted therapies become available, the development of increasingly adding cancer genome and transcriptome sequencing to regular clinical practice might help in this systematic approach of targeting signalling networks most relevant to individual tumours. However, clonal diversity can severely complicate this class of analysis (see Section 3.4). Additionally, signalling networks perturbed in one way can rewire elsewhere and confer resistance, indicating a challenge in providing straightforward biomarkers for response for targeted therapies (Prahallad *et al*., 2012). Therapies combining multiple pathway inhibitors are being tested as a way to prevent resistance to individual drugs, but these strategies may face important toxic effects on normal tissues that limit their implementation.

### 5.2 Therapies against cancer stem cells

The hierarchical organization of CRC has led to the hypothesis that the cause of disease relapse is that standard chemotherapy eliminates the tumour bulk while sparing CRC‐SCs. While formal proof for this idea is still lacking, several initiatives to develop anti‐CRC‐SC therapies are currently ongoing. There are three classes of such therapies. First, targeting the key pathways that regulate the behaviour of CRC‐SCs – including WNT, NOTCH, EGFR and TGF‐beta/BMP – is an obvious approach to prevent the maintenance or expansion of this cell population. Whereas inhibitors and agonist of these pathways exist, they are not always effective, given the fact that CRCs carry genetic alterations that alter many or all of these pathways. For instance, CRC‐SCs depend on WNT signalling to sustain self‐renewal, and inhibitors of WNT secretion and of WNT receptors are in advanced stages of testing. Yet, the vast majority of CRCs carry mutations in the tumour suppressor gene *APC*, which constitutively activate the pathway downstream of the receptor. Unfortunately, developing inhibitors that target pathway components downstream of APC has proven to be tremendously challenging (Anastas and Moon, 2012; Kahn, 2014), although two recent studies reported promising compounds. The first, called NCB‐0846, inhibits TNIK (an essential regulatory component of WNT/beta‐catenin signalling (Mahmoudi *et al*., 2009)) and effectively abrogates CRC stemness *in vitro* and polyp formation in mice (Masuda *et al*., 2016). The second (called MSAB) binds to beta‐catenin and promotes its proteasomal degradation, inhibiting the growth of xenograft tumours in mice (Hwang *et al*., 2016) (Fig. 2). A drawback of this class of strategies is the fact that normal ISCs also critically depend on the same pathways as CRC‐SCs, which may lead to strong side effects.

A second therapeutic strategy is to deplete CRC‐SCs through the use of antibodies–drug conjugates (ADCs) designed to bind surface antigens expressed by this cell population. As recently shown, antibodies targeting Lgr5 coupled to different toxins demonstrated potent antitumour efficacy in preclinical models of CRC (Junttila *et al*., 2015) (Fig. 3). Again, this strategy may also have significant side effects given the fact that many surface makers of CRC‐SCs are shared with normal ISCs. Furthermore, as discussed in Section 3.3, if stress‐induced dedifferentiation in the normal crypt has its parallel in cancer, regeneration of CRC‐SCs pool by conversion of non‐SCs cells upon cessation of the treatment may also limit the effectiveness of this strategy (Fig. 3).

A third strategy relies on identifying molecules and dependencies specific for CRC‐SCs that can be targeted therapeutically, supposedly without major toxicities. For instance, targeting self‐renewal of CRC‐SCs using an inhibitor of BMI1 (PTC‐209) has been shown to have robust therapeutic effects (Kreso *et al*., 2014). ER‐stress‐induced activation of the unfolded protein response (UPR) forces CRC‐SCs to differentiate, and therefore, drugs that induce UPR could have therapeutic activity against this cell population (Wielenga *et al*., 2015). Furthermore, a low dose of the DNA‐demethylating agent 5‐AZA‐CdR induces viral‐like response in CRC‐SCs by triggering the expression of double‐stranded RNAs derived from endogenous retroviral elements, which has an antitumoral effect (Roulois *et al*., 2015) (Fig. 3).

### 5.3 Stromal therapies

In line with the growing realization that a large number of stromal cells are actively involved in driving CRC progression (including maintenance of CRC‐SCs), new strategies are taking shape wherein either the TME is the main target or a combination of agents attacks both cancer cells and stromal cells, to improve therapeutic response and prevent acquired resistance (Fang and Declerck, 2013). For example, a recent study suggested that HGF secreted by fibroblasts might decrease the response to irinotecan. The successful reversion of such resistance using anti‐HGF‐targeted therapy (Woo *et al*., 2015) emphasizes the promises of multitargeted treatments for patients (Fig. 3). This strategy might also be of high value for CRC patients with nonmutated KRAS gene where resistance to inhibitors of EGFR is associated with increased levels of HGF (Liska *et al*., 2011; Takahashi *et al*., 2014).

As mentioned in Section 4.2, another group of widely applied agents for CRC treatment in fact target the TME. These drugs inhibit VEGF signalling in endothelial cells and thereby oppose tumour vascularization. Besides monoclonal antibodies, also a recombinant fusion protein (aflibercept, blocking VEGFA, VEGFB and placental growth factor signalling) was shown to improve survival in phase III trials on selected patients with metastatic CRC (Van Cutsem *et al*., 2012). However, the majority of responsive patients tend to develop resistance to anti‐angiogenic therapies over time*,* in part because the hypoxic conditions established during treatment may independently cause further malignization of cancer cells (Ulivi *et al*., 2016). Nevertheless, anti‐angiogenic therapies are likely to continue to be part of future combinatory treatment strategies.

Instead of targeting elements in the TME for depletion or destruction, an attractive alternative is the repolarization of stromal cells into a nonpermissive state, for instance by blocking or reverting the corruptive signals from cancer cells. In this perspective, stromal reprogramming might have a lower toxicity than destructive therapies and can thus be a powerful tool to combine with more conventional treatments. The many stromal pro‐tumorigenic functions associated with TGF‐beta in CRC (and other cancers) suggest that its successful and safe inhibition would be an invaluable therapeutic goal. In a mouse model of metastatic initiation, stromal TGF‐beta signalling enhanced metastatic spreading, while therapeutic inhibition of this pathway in the stroma with galunisertib abrogated liver metastasis initiation by CRC cells (Calon *et al*., 2012, 2015) (Fig. 3). Extensive discussion on the implementation of anti‐TGF‐beta therapies for advanced CRC can be found elsewhere (Tauriello and Batlle, 2016).

### 5.4 Immunotherapies

It has been proposed that conventional chemotherapy may in large part rely on immune components to be efficient, for instance by causing immunologic cell death (Zitvogel *et al*., 2013). Consequently, T‐cell infiltration of both the primary tumour and liver metastases has been associated with response to chemotherapy in patients with metastatic CRC (Halama *et al*., 2009, 2011). Immunotherapies – which aim to directly induce immune responses, or to enhance pre‐existing ones – have seen impressive efficacies in a growing number of cancer types (Palucka and Coussens, 2016). Designed to activate tumour‐specific CD8^+^ CTL immune responses, cancer vaccines have demonstrated benefit in prostate cancer, melanoma and other cancer types (Butterfield, 2015), and several strategies have been developed for CRC (Xiang *et al*., 2013). Many such treatments focussed on advanced (metastatic) disease have had disappointing responses, possibly in part because of the progressively immunosuppressive TMEs of those tumours, suggesting a higher benefit at earlier stages (Merika *et al*., 2010). Recently, promising results were reported for a vaccine based on a mucin‐1 peptide in prophylactic treatment of patients with precancerous adenomas (Kimura *et al*., 2013). Even in this study, treatment failure was linked to the presence of high levels of immunosuppressive MDSCs already at this early stage, which might be a useful biomarker for further exploration of similar strategies. Furthermore, these cells can be therapeutic target themselves. To circumvent both the requirement of *in situ* activation and the problem of tumoral immune tolerance, a passive form of immunotherapy can be used, where *in vitro*‐activated immune effectors (most often T cells) are administered to the patient. However, early trials with adoptive cell therapy resulted in severe toxicities and were not efficacious (Xiang *et al*., 2013).

Early clinical trials with a different type of immunotherapy – checkpoint inhibition, which unblocks T cell‐mediated adaptive anticancer responses – have shown benefit in at least a subset of patients with CRC (Puzzoni *et al*., 2016; Zumwalt and Goel, 2015) (Fig. 3). Notably responsive are hypermutated MSI tumours, which commonly carry many neoantigens, are heavily infiltrated by T lymphocytes and express relatively high levels of various checkpoints (Diaz and Le, 2015; Kloor *et al*., 2010; Llosa *et al*., 2015). However, even in microsatellite‐stable CRCs, there is a correlation between mutational/neoantigen load, immune infiltration and survival (Giannakis *et al*., 2016), offering a perspective on successful future exploitation of immunotherapies. Several clinical trials are ongoing, evaluating the benefit of checkpoint inhibitors such as anti‐CTLA‐4 or anti‐PD‐1 antibodies (Moehler *et al*., 2016). In addition, combinations of multiple checkpoint inhibitors, or of such agents with other strategies such as vaccines and/or chemotherapy, are likely to increase the number of patients with good responses (Sharma and Allison, 2015).

Alternatively, immunotherapy can be designed to inhibit pro‐tumorigenic interactions between immune cells and neoplastic CRC cells. In a phase I trial, cancer–stromal crosstalk through accumulating myeloid cells and T cells, and pro‐tumorigenic cytokine signalling, was successfully targeted using anti‐CCR5 therapy in patients with advanced/metastatic CRC (Halama *et al*., 2016) (Fig. 3). As TGF‐beta is a classical immune suppressor as well as a key modulator of cellular crosstalk, the discovery that high levels of TGF‐beta correlate with poor prognosis may imply that colorectal cancer exploits this cytokine in tumoral immune evasion, besides affecting CAF‐mediated secretion of pro‐tumorigenic factors (Tauriello and Batlle, 2016). It will be of great interest to study the effects of this therapeutic strategy in immunocompetent models, as well as explore the putative role of CAFs as immunosuppressors (Feig *et al*., 2013; Kraman *et al*., 2010). Indeed, TGF‐beta inhibition, for which several approaches are in clinical trials (Akhurst and Hata, 2012; Neuzillet *et al*., 2015; Smith *et al*., 2012), might act as or synergize with immunotherapy.

## 6 Concluding remarks

Taken together, recent data discussed here emphasize the importance of tumour heterogeneity – in terms of cellular hierarchy, clonal diversity and tumour microenvironment – in modulating CRC progression and metastasis (Fig. 1). These factors have strong implications for patient stratification as well as for the development, optimization and application of therapeutic strategies (Figs 2 and 3). While key mechanisms and dependencies in cancer progression and metastasis are increasingly being translated into targeted therapies, it is vital to integrate these emerging concepts both in the selection of patients for clinical testing of new agents and in combining approved therapies for the treatment of individual patients.

All types of heterogeneity play a role in generating and maintaining colorectal cancer stem cells (CRC‐SCs) with chemoresistance and metastatic competency, and particularly, the TME supports metastatic colonization (Figs 2 and 3). Therefore, therapies that target any or several of the mechanisms discussed here may potentially be able to prevent metastasis from developing in the 40–50% of patients with early‐stage disease at risk of distant recurrence. Moreover, several of these therapies may be very beneficial for patients with advanced/metastatic CRC. Among these promising therapeutic strategies are treatments that target CRC‐SCs directly or through their dependency on the TME. In addition, a deeper understanding of colorectal cancer immunity may lead to a better exploitation of immunotherapeutic options, as well as offering opportunities in targeting immunosuppressive mechanisms.

The fast pace of progress as well as the high number of open questions in each of the research fields we have borrowed from assures that there will be significant challenges ahead in our understanding of the full complexity of CRC as a heterogeneous disease. Breaking our inability to effectively treat metastasis requires the concerted effort of a large number of researchers and clinicians and will likely involve patient‐specific combination of therapies aimed at targeted elimination of metastatic competency mechanisms.
